# Liver Atrophy Associated With Monolobar Caroli's Disease

**DOI:** 10.1155/1991/29763

**Published:** 1991

**Authors:** L. N. Mohan, P. G. Thomas, A. B. Kilpadi, S. D'Cunha

**Affiliations:** Unit of Hepatobiliary and Pancreatic Surgery Department of Surgery St. John's Medical College Hospital Bangalore 560 034 India

## Abstract

The association of the atrophy-hypertrophy complex in monolobar Caroli’s disease (Type I) is reported in a 30 year old male who presented with recurrent cholangitis. Ultrasound and CT scan showed localised, right sided, saccular biliary dilatation in a normal sized liver. Severe right lobar atrophy was detected at operation and the resected right lobe weighed only 140 gms. Distortion of the hilar vascular anatomy and posterior displacement of the right hepatic duct orifice were problems encountered at surgery.


HPB Surgery, 1991, Vol. 4, pp. 203–207
Reprints available directly from the publisher
Photocopying permitted by license only

© 1991 Harwood Academic Publishers GmbH
Printed in the United Kingdom

### LIVER ATROPHY ASSOCIATED WITH MONOLOBAR CAROLI'S DISEASE

L.N. MOHAN, P.G. THOMAS, A.B. KILPADI and S. D'CUNHA
*Unit of Hepatobiliary and Pancreatic Surgery, Department of Surgery, St. John's Medical College Hospital, Bangalore 560 034, India*

*(Received 22 January 1991)*

The association of the atrophy-hypertrophy complex in monolobar Caroli's disease (Type I) is reported in a 30 year old male who presented with recurrent cholangitis. Ultrasound and CT scan showed localised, right sided, saccular biliary dilatation in a normal sized liver. Severe right lobar atrophy was detected at operation and the resected right lobe weighed only 140 gms. Distortion of the hilar vascular anatomy and posterior displacement of the right hepatic duct orifice were problems encountered at surgery.

KEY WORDS: Caroli's disease, liver atrophy, bile ducts, CT

### INTRODUCTION

The unique characteristics of Caroli's disease continue to be described in the literature. Its association with recurrent cholangitis, intrahepatic stones, epithelial dysplasia, and malignancy are well documented [1,2]. Unilobar and generalised forms are known, and may be associated with congenital hepatic fibrosis and portal hypertension [3,4]. Severe atrophy of the liver in unilobar Caroli's disease has not been previously highlighted and could be an important consideration while planning surgical treatment.

### CASE REPORT

C., a 30 year old male, presented with episodes of recurrent cholangitis since childhood. Ultrasound scan done at another hospital had shown dilated intrahepatic bile ducts. ERCP was performed with a diagnosis of Caroli's disease, but failed to opacify the dilated ducts. The extrahepatic biliary tree was normal.

When seen at our hospital, the patient was anicteric, and on abdominal examination no abnormality was detected. Liver function tests were normal except for a mildly elevated alkaline phosphatase level of 318 U/L (N up to 270 U/L). Renal function was normal. Abdominal ultrasound showed intrahepatic ductal dilatation localised to the right lobe. The intrahepatic portal structures were

Address correspondence to: Dr. Philip G. Thomas, Assistant Professor, Department of Surgery, St. John's Medical College Hospital, Bangalore 560 034, India.

normal, but the right hepatic vein could not be delineated. CT scan was performed, and revealed gross intrahepatic ductal dilatation confined to the right lobe, with the rest of the liver being of normal size and texture. The kidneys and other viscera were normal (Figure 1).

Right hepatic lobectomy was performed. On dissection of the porta and occlusion of the right hepatic artery and right portal vein the line of demarcation revealed the right lobe to be severely atrophic. Compensatory hypertrophy of the left lobe, particularly segment IV had resulted in displacement of the gallbladder to the right border of the liver, with the entire right liver shrunken and displaced posteriorly. Rotation of the hilar structures had caused the right hepatic duct to course in a postero-anterior direction. The duct itself showed cystic dilatation, and was filled with muco-pus and debris, which had occluded its opening into the common hepatic duct. The portal vein was displaced to the left and its right branch was situated posterior to the left hepatic duct.

The excised right liver weighed only 140 gms (Figure 2) and on section showed grossly dilated bile ducts. Microscopic examination showed severe nonspecific cholangitis. The liver parenchyma surrounding the cystic spaces showed hepatocytes filled with yellow brown pigment with a chronic inflammatory infiltrate. Periportal fibrosis was not present.

Except for a transient bile leak from the stump of the right hepatic duct, which settled with nasobiliary drainage, the patient made an uneventful recovery and on follow up at four months is doing well.

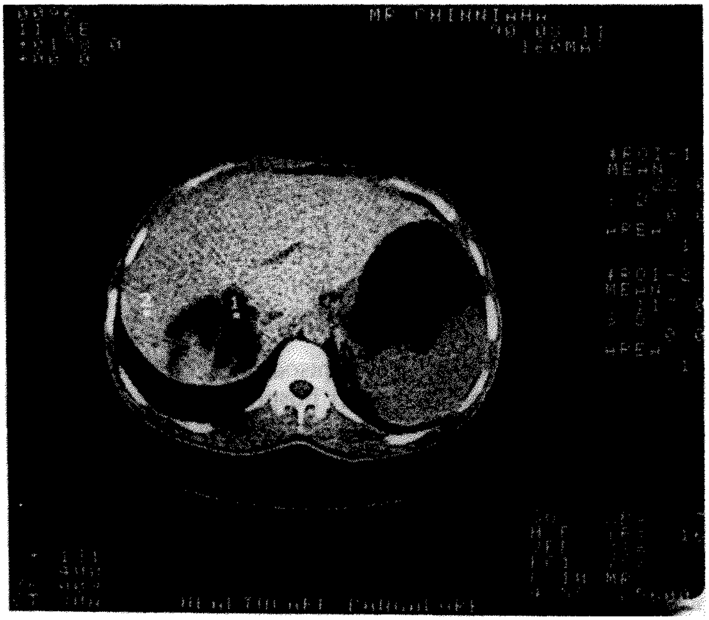

**Figure 1** CT scan showing saccular dilatations of intrahepatic ducts (1) within the right liver lobe (2).

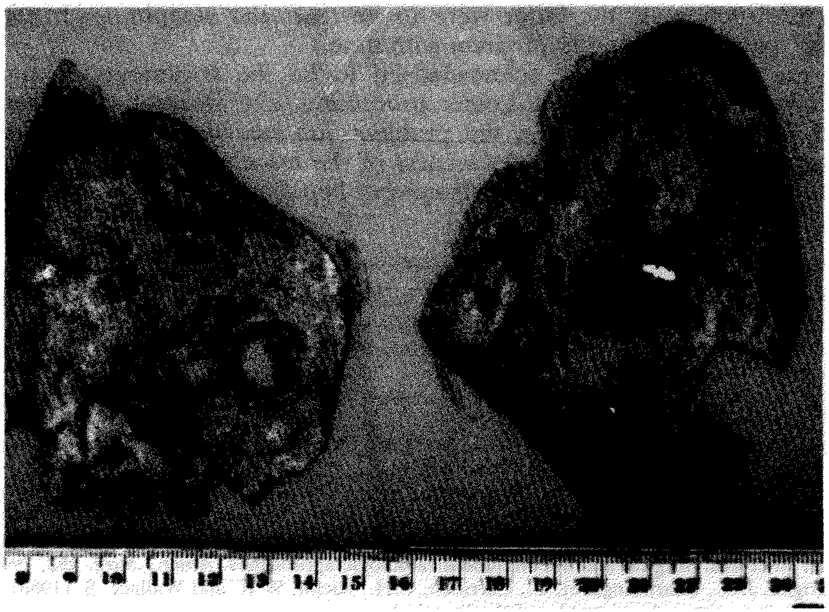

**Figure 2** Cut section of the resected right lobe showing atrophy (Wt. 140 Gms.) and dilated bile ducts.

### DISCUSSION

Caroli's disease, defined as congenital segmental saccular dilatation of the intrahepatic bile ducts, is now recognised to occur in two distinct forms: the simple type, and the periportal fibrosis type [4,5]. The simple type is rarer, and usually presents in adult life with recurrent cholangitis dating back to childhood, whereas the second type — similar to if not identical with congenital hepatic fibrosis — presents with features of portal hypertension [2,3].

The occurrence of the atrophy-hypertrophy complex in this disease has not been emphasised in the literature, and may be of special interest to the surgeon contemplating resection, as the association of this complex with distortion of the anatomy of the portal structures is well recognised [6]. Severe atrophy of the right hepatic lobe resulting in lateral migration of the gallbladder due to compensatory hypertrophy of the left lobe has been described in association with tumours [7], and benign bile duct strictures [6,8]. In two of six cases of simple, or Type I, Caroli's disease Nagasue reported marked atrophy of the involved liver lobe noticed at operation. However, three patients had some degree of atrophy detected on radioisotope scanning of the liver[5].

Experimental studies in dogs have shown that atrophy of the liver is associated with biliary obstruction [8], and may be the result of decreased blood flow to the involved segments [9]. Liver atrophy may also accompany primary hepatocellular disease unassociated with bile duct obstruction[10]. In Caroli's disease, the question is whether atrophy is a primary association or whether it is secondary to prolonged biliary obstruction. If the latter were to be true, the atrophy — hypertrophy complex may be expected to progress with time.

Hepatic resection is now acknowledged to be the treatment of choice for monolobar Caroli's disease[3,5]. Lesser procedures like internal drainage of the dilated ducts fail to overcome the problems of recurrent cholangitis and the development of malignancy [11]. Awareness of the association of lobar atrophy, as herein reported, would help in the planning of hepatic resection for this condition.

### *References*

1. Marchal, G.J., Desmet, V.J and Proesmans, W.C. *et al.* (1986) Caroli's Disease: High frequency US and pathologic findings. *Radiology*, **158**, 507–511
2. Dayton, M.T., Longmire, W.P. and Tompkins, R.K. (1983) Caroli's disease : A premalignant condition? *Amer.J.Surg.* **145**, 41–48
3. Mercadier, M., Chigot, J.P., Clot, J.P., Langlois, P. and Lansiaux, P. (1984) Caroli's disease. *World J.Surg.* **8**, 22–29
4. Hoglund, M., Muren, C. and Schmidt, D. (1989) Caroli's disease in two sisters. Diagnosis by ultrasonography and computed tomography. *Acta Radiol.* **30**, 459–462
5. Nagasue, N. (1984) Successful treatment of Caroli's disease by hepatic resection. Report of six patients, *Ann. Surg.* **200**, 718–723
6. Czerniak, A., Soreide, O. and Gibson, R.N. *et al.* (1986) Liver atrophy complicating benign bile duct strictures. Surgical and interventional radiologic approaches. *Amer.J.Surg.* **152**, 294–300
7. Lorigan, J.G., Charnsangavej, C., Carrasco, C.H., Richli, W.R. and Wallace, S. (1988) Atrophy with compensatory hypertrophy of the liver in hepatic neoplasms: Radiographic findings. *A.J.R.* **150**, 1291–1295
8. Braasch, J.W., Whitcombe, F.F., Watkins, E., Maguire, R.R. and Khazei, A.M. (1972) Segmental obstruction of the bile duct. *Surg.Gynecol.Obst.* **134**, 915–920
9. Aaronsen, K.F., Nylander, G. and Ohlsson, E.G. (1969) Liver blood flow studies during and after various periods of total biliary obstruction in the dog. *Acta Chir.Scand.* **135**, 55–59
10. Ham, J.M. (1979) Partial and complete atrophy affecting hepatic segments and lobes. *Br.J.Surg.* **66**, 333–337
11. Watts, D.R., Lorenzo, G.A. and Beal, J.M. (1974) Congenital dilatation of the intrahepatic biliary ducts. *Arch.Surg.* **108**, 592–598

*(Accepted by S. Bengmark 30 January 1991)*

### INVITED COMMENTARY

A pitfall in the diagnosis of Caroli's disease is apparently a lack of awareness of the disease, as is easily recognized by reviewing the world literature, unnecessary or fruitless operations are not infrequently performed or repeated. Physicians should always consider the possibility of Caroli's disease when a patient has the typical symptom of recurrent pain, fever, and/or jaundice from his or her childhood. Surgeons should investigate the liver when they can not find any pathology in the extrahepatic biliary system in a patient who has presented with a typical clinical manifestation of gallstones.

Important therapeutic points in Caroli's disease are to relieve disabling clinical symptoms such as pain, fever, or jaundice and to cure or present cholangitis and liver abscess, which may lead to life-threatening sepsis. External drainage or bilio–digestive anastomoses are widely used but these methods are usually not very effective. Liver resection may be most effective for unilobar or segmental lesions of type I disease. However, two points should be borne in mind when one performs such resections. The first is that the liver is often distorted due to scar formation and/or regional atrophy as in the present case, and surgeons should be very careful not to injure the main vessels and ducts of the remaining liver, particularly at the liver hilus. The second is that not only extensive pre- and postoperative chemotherapy with broad-spectrum antibiotics but careful operative technique may be important to reduce or eliminate infective complications since dilated ducts are frequently infected irrespective of the patient's preoperative symptoms and laboratory data.

The best treatment for type II disease and intractable type I disease may be hepatic transplantation.

Naofumi Nagause
Associate Professor
Second Department of Surgery
Shimane Medical University
Izumo 693, Japan

